# Editorial for the Special Issue: Gut Microorganisms of Aquatic Animals

**DOI:** 10.3390/microorganisms7100377

**Published:** 2019-09-21

**Authors:** Konstantinos Ar. Kormas

**Affiliations:** Department of Ichthyology & Aquatic Environment Faculty of Agricultural Sciences, University of Thessaly, Volos 382 21, Greece; kkormas@uth.gr

Since the introduction of the term holobiont [1], the scientific interest on the associations between microbes and various hosts—namely plants, animals, and other microbes—boomed, especially the last decade. Indeed, the investigation of the microbe–host associations has stemmed a truly interdisciplinary science, whereby hypotheses are generated and answered from groups of distant disciplines at first sight. Today, we know that practically all investigated animal organisms harbor diverse and multifunctional microbiota. However, as such complex systems remain elusive for a full description of their biological relations, the first step, that of discerning which microbe is found on or in which tissue of a specific animal, remains essential to deepen our knowledge on holobionts. Taking into account the hundreds of thousands to millions of prokaryotic cell abundance in the aquatic environment, it is realistic for aquatic animals, along with their associated microbiota, to be seen as microbial ecosystems swimming in seas, lakes or rivers of microbes. Moreover, as we get a sharper insight on these associations, symbiotic microbes can reciprocally interact with the aquatic environment even at the ecosystem level [2,3]. The articles published in the special issue “Gut Microorganisms of Aquatic Animals” are a contribution towards the scientific efforts to unravel some of the associations between freshwater [4], ornamental [5] and marine [6,7,8] fish, as well as shrimp [9,10,11] and their microbes. These papers cover multiple issues on aquatic animal–microbe interactions: healthy or diseased fish, the impact of pollution and handling practices on fish gut microbiota, and the effect of probiotics in the gut. Such associations are now widely recognized as highly important to aquaculture [12,13].
